# High-Pulse-Repetition-Rate Eye-Safe Raman Laser with Acousto-Optic Q-Switched Device

**DOI:** 10.3390/mi16020222

**Published:** 2025-02-16

**Authors:** Yu-Hsin Hsu, Song-Qing Lin, Dai-Jun Liu, Hsing-Chih Liang, Yung-Fu Chen

**Affiliations:** 1 Department of Electrophysics, National Yang Ming Chiao Tung University, Hsinchu 30010, Taiwan; yuhsin.sc10@nycu.edu.tw (Y.-H.H.); songqinglin634.sc11@nycu.edu.tw (S.-Q.L.); daijunliu4403112.sc12@nycu.edu.tw (D.-J.L.); 2 Institute of Physics, National Yang Ming Chiao Tung University, Hsinchu 30010, Taiwan; hcliang@nycu.edu.tw

**Keywords:** acousto-optic, Q-switch, eye-safe laser, Raman scattering

## Abstract

The acousto-optic Q-switch is exploited to develop a high-repetition-rate eye-safe Raman laser at 1526 nm. The Nd:YVO_4_ and KGW crystals are employed as the fundamental laser and Stokes Raman gain materials, respectively. The influence of the gate-open time on the performance is systematically explored for the repetition rate between 80 and 150 kHz. The separate configuration is used to construct the resonant cavities for the fundamental and Stokes waves to achieve a pulse width that is as short as possible. Under the optimal alignment, the average output power can exceed 5.0 W at a pump power of 30 W for a repetition rate within 100–150 kHz with a gate-open time of 0.5 μs. In addition, the output peak power can be greater than 10 kW for a pulse repetition rate between 80 and 120 kHz. The optical-to-optical conversion efficiency is up to 16.7%, which is better than that obtained by the Nd:YVO_4_/YVO_4_ system.

## 1. Introduction

**Figure 1:**
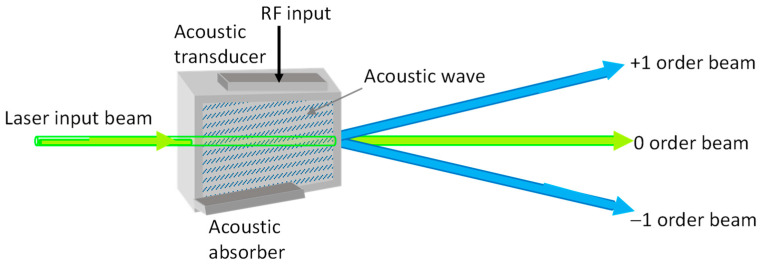
Basic situation for acousto-optic Q-switched device.

Acousto-optic modulators (AOMs) have been widely used for controlling the power, frequency, or spatial direction of a laser beam with an electrical drive signal. The acousto-optic Q-switches are a type of AOM for implementing actively Q-switched solid-state lasers. The fundamental principle of the acousto-optic Q-switch is that it exploits the interaction between an acoustic wave and a light beam in a scattering material. When an RF signal is impressed to the piezoelectric transducer adhered to the molten quartz, this causes an acoustic shear wave to form phase grating. The light beam is diffracted when it satisfies the Bragg angle with respect to this phase grating, and is separated in space from the incident beam. Figure 1 simply shows the basic situation for an acousto-optic Q-switched device. For Q-switched solid-state lasers, the zero-order beam is usually used to construct the cavity under lasing conditions. In other words, when a radio frequency (RF) signal is injected into the Q-switched device, the diffracted beam deviates from the optical axis of the laser cavity. At the beginning of pumping, the acousto-optic Q-switch generates the modulation loss to cause the laser cavity to remain in a low quality-value (Q-value) stage, preventing laser oscillation. Meanwhile, a large amount of inversion population can be accumulated in the upper energy level. When the RF signal is suddenly reduced to zero within an interval of the gate-open time *T*_go_ to switch the cavity to the high Q-value state, the accumulated energy is rapidly activated as laser oscillation, resulting in high-peak-power giant pulse laser output. Q-switched solid-state lasers have been extensively employed in a variety of applications including material processing [1], micromachining [2], remote sensing [3,4], optical-resolution photoacoustic microscopy [5], and stimulated emission depletion microscopy [6].

Laser sources within the spectral region between 1.4 and 1.8 μm are called eye-safe lasers because they are strongly absorbed by the vitreous body before reaching the retina. As a consequence, light sources within 1.4–1.8 μm are of intense interest for numerous applications in range finding, scanning lidar, remote sensing, and laser countermeasures [3,4]. In addition, laser sources with a spectrum near 1.5 μm are particularly low-loss in the transmission of optical fibers and atmosphere, which allows them to have useful applications in optical fiber communications [7]. Furthermore, Q-switched eye-safe lasers are highly desirable for low-altitude navigation, targeting infrared for night, and mid-infrared laser generation [8]. Q-switched lasers with a repetition rate higher than 100 kHz are highly desirable for high-speed scanning [9,10,11,12,13].

Currently, technologies for the production of eye-safe lasers include erbium-doped lasers [14,15], semiconductor lasers [16,17], and solid-state lasers with optical parametric oscillators (OPOs) [18,19,20] or stimulated Raman scattering (SRS) [21,22,23,24,25,26,27]. Raman scattering is an inelastic scattering process in which incident photons interact with molecules, gaining or losing energy, resulting in a change in the frequency of the scattered photons. The increasingly broad applications in various fields have created a huge demand for the development of reliable all-solid-state lasers in previously undiscovered spectral regions. Compared with gaseous and liquid Raman cells, the advantages of SRS in solid-state crystals include higher conversion efficiency, no need for phase matching, and easier operation [28,29]. In particular, the high damage thresholds of crystalline Raman lasers can be used to obtain Q-switched eye-safe lasers with high average power, high peak power, and high beam quality. Considerable progress has been made so far in eye-safe crystalline Raman lasers in the nanosecond pulse range. One of the promising Raman materials is the YVO_4_ crystal. In 2004, a Nd:YVO_4_ crystal was firstly used to create an eye-safe self-Raman laser via converting the fundamental wave at 1342 nm to the Stokes wave at 1525 nm [30]. In 2009, a double-end diffusion-bonded Nd:YVO_4_ crystal was designed to develop an eye-safe self-Raman laser with average output power achieving 2.23 W [31]. Even though the self-Raman scheme is compact and efficient, power scaling-up is hindered by the two-fold thermal loading, i.e., the energy difference between the pump and the fundamental photons and that between the fundamental and the Stokes photons. In order to reduce the thermal load, the separation of the laser and Raman crystals was proposed in 2014 to construct a Nd:YVO_4_/YVO_4_ eye-safe laser at 1525 nm, obtaining a peak power of 3.4 kW at a repetition rate of 140 kHz [32]. In 2024, a Nd:YVO_4_/YVO_4_ eye-safe Raman laser was improved to achieve a peak power of 25 kW at a repetition rate of 100 kHz [33]. Another promising Raman gain medium is the potassium gadolinium tungstate KGd(WO_4_)_2_ or KGW single crystal due to its low structure symmetry and high nonlinearity. In addition, KGW crystals have a strong Raman spectrum extending from the ultraviolet to the near-infrared, so they can be used as reference materials for Raman spectroscopy as well as samples for studying Raman scattering in solids. In 2023, a high-beam-quality nanosecond pulse intracavity KGW Raman laser operating at 1526 nm was constructed by using a V-shaped resonant cavity and a composite Nd:YVO_4_ crystal, obtaining an average output power of 4.9 W and a peak power of 13 kW [34]. Nevertheless, the highest repetition rate for this eye-safe Raman laser was merely 25 kHz. In addition to Nd:YVO4 material, Nd:YLF crystals are usually used to develop high-pulse-energy low-repetition-rate eye-safe lasers [35].

In this work, we demonstrate a high-repetition-rate acousto-optic Q-switched Nd:YVO_4_/KGW eye-safe Raman laser at 1526 nm. We thoroughly investigate the influence of the gate-open time on the laser performance for a repetition rate from 80 to 150 kHz. In order to shorten the pulse width, a configuration of separate cavities is employed for the fundamental and Stokes waves. One end surface of the KGW Raman medium is coated to form the dichroic mirror in order to lower the various losses for the Stokes wave. Under the optimal condition, the average output power can generally exceed 5.0 W at a pump power of 30 W for a repetition rate within 100–150 kHz. Under the same circumstances, the pulse width is generally shorter than 5.4 ns. The overall optical-to-optical conversion efficiency is greater than 16.7%.

## 2. Experimental Setup

**Figure 2:**
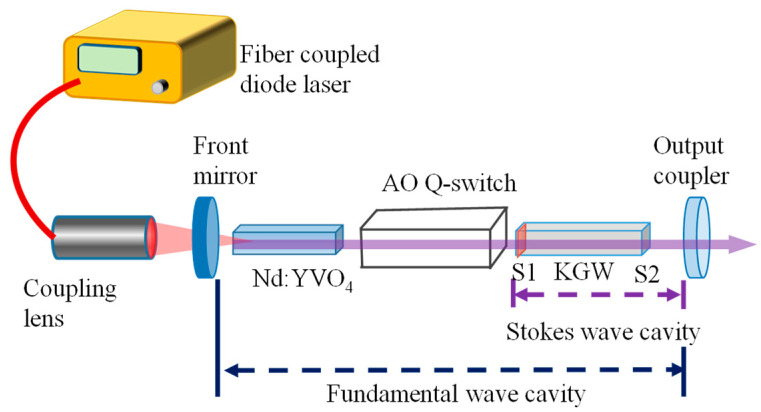
Experimental configuration for implementing the acousto-optic Q-switched eye-safe Nd:YVO_4_/KGW Raman laser at 1526 nm.

Figure 2 shows the experimental configuration for the implementation of the acousto-optic Q-switched eye-safe Nd:YVO_4_/KGW Raman laser at 1526 nm. The laser resonator was designed to comprise a symmetrical concave–concave cavity for the fundamental wave at 1342 nm and a coupled plano–concave cavity for the Raman Stokes wave at 1526 nm. The light source for pumping the Nd:YVO_4_ crystal was a fiber-coupled diode laser with a central wavelength around 808 nm and a maximum output power of 30 W. The specifications of the coupling fiber for the pump diode were 200 μm for the core diameter and 0.22 for the numerical aperture. By using the re-imaging lenses, the pump source was focused on the Nd:YVO_4_ crystal with a pump diameter of approximately 500 μm. The specifications of the Nd:YVO_4_ crystal were a-cut with 0.2 at.% in dopant concentration and dimensions of 3 × 3 × 15 mm^3^. The Nd:YVO_4_ crystal was coated to be antireflective (AR, reflectance < 0.5%) on both end surfaces for the spectral range of 800–1400 nm. A N_p_-cut KGW material with dimensions of 3 × 3 × 20 mm^3^ was exploited as the Raman crystal. The polarization of the fundamental wave was placed along the N_m_ axis of the KGW crystal to generate the Stokes wave near 1526 nm from the fundamental wave at 1342 nm via a Raman shift of 901 cm^−1^. For the separate configuration of the laser resonators, the facet S_1_ of the KGW crystal toward the Nd:YVO_4_ crystal had a dichroic coating with high transmission (HT, transmittance > 99.0%) at 1342 nm and high reflection (HR, reflectance > 99.5%) at 1526 nm. The second facet S_2_ had an AR coating (reflectance < 0.5%) within 1300–1600 nm. It is worthwhile to mention that the dichroic coating on the facet of the KGW Raman crystal can effectively reduce the scattering losses coming from an additional intracavity mirror. Without the dichroic coating on the facet of the KGW crystal, an additional intracavity mirror was required to constitute an HR mirror for the SRS resonator [36]. Both the laser and Raman active crystals were enclosed with indium foils and packaged in copper holders with temperature control at 20 °C by conduction cooling. The input mirror of the resonator was a concave mirror with a radius of curvature of 100 mm. The plano side of the input mirror was coated to be AR (reflectance < 0.2%) at 808 nm. The concave side for forming the resonator had a dichroic coating with HT (transmittance > 95%) at 808 nm, HT (transmittance > 85%) at 1064 nm, and HR (reflectance > 99.9%) at 1342 nm. The output coupler was also a concave mirror with a radius of curvature of 100 mm. The concave side of the output coupler for forming the resonator had a coating with HT (transmittance > 85%) at 1064 nm, HR (reflectance > 99.9%) at 1342 nm, and partial reflection (PR) (reflectance ≈ 80%) at 1526 nm. The other surface of the output coupler had an AR (reflectance < 0.2%) coating at 1526 nm.

The acousto-optic Q-switcher (Gooch & Housego, Ilminster, UK) was driven by a 40.68 MHz RF generator to be operated at 20 W. The length of the acousto-optic Q-switcher was 30 mm, and both end surfaces had an AR (reflectance < 0.5%) coating at 1342 nm. The temporal characteristics of the output Q-switched pulses were detected with a high-speed InGaAs photodetector (Electro-optics Technology Inc., Traverse, MI, USA, ET-3500 with rise time < 25 ps). The signals for the pulse width and peak-to-peak stability were measured using a digital oscilloscope (Teledyne LeCroy, Chestnut Ridge, NY, USA, Wave Master 820Zi-A) with sampling rates of 25 ps and an electrical bandwidth of 20 GHz. The optical spectrum of the laser output was analyzed with a resolution of 0.1 nm (Advantest Q8381A, Chiyoda, Japan).

## 3. Experimental Results and Discussion

The mode size of the fundamental wave in the Raman crystal can be reasonably computed with the cavity mode radius at the beam waist of the fundamental wave, which is given by

(1) $$\omega_{F}={\left[\frac{\lambda_{F}}{\pi }\sqrt{L_{cav}^{*}(2R-L_{cav}^{*})}\right]}^{1/2}$$

where $L_{cav}^{*}$ is the optical length of the fundamental wave cavity, *R* is the radius of curvature of the cavity mirrors and *λ_F_* is the wavelength of the fundamental wave. On the other hand, the mode size of the Stokes wave in the Raman crystal can be computed with the cavity mode radius at the beam waist of the Stokes wave, which is given by

(2) $$\omega_{R}={\left[\frac{\lambda_{R}}{2\pi }\sqrt{L_{SRS}^{*}(R-L_{SRS}^{*})}\right]}^{1/2}$$

where $L_{SRS}^{*}$ is the optical length of the Stokes wave cavity and *λ_R_* is the wavelength of the Stokes wave. The experimental results clearly revealed that the shorter the cavity length, the higher the output efficiency. Accordingly, the cavity lengths for $L_{cav}^{*}$ and $L_{SRS}^{*}$ were set to be as short as possible, at 68 and 20 mm, respectively. The cavity mode sizes for $\omega_{F}$ and $\omega_{R}$ were calculated as 0.185 and 0.121 mm, respectively.

**Figure 3:**
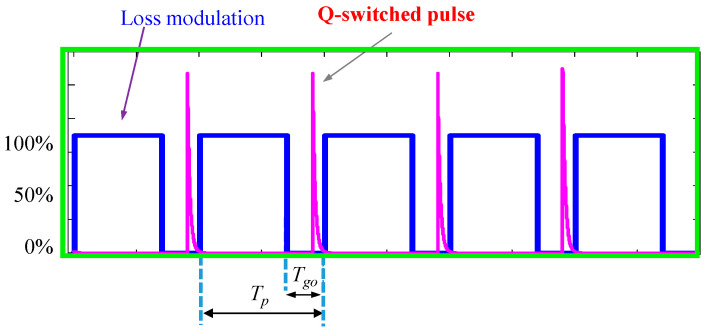
Temporal characteristics of the acousto-optic modulation loss and laser output pulse.

Figure 3 qualitatively depicts the temporal characteristics of the acousto-optic modulation loss and laser output pulse for the operation of the Q-switched laser. The pulse buildup time *T*_bu_ specified in Figure 3 represents the time delay between the emission of output pulse and the shutting down of acousto-optic modulation losses. For acousto-optic Q-switched Nd-doped solid-state lasers with cavity lengths around several tens of centimeters, the pulse buildup time *T*_bu_ was generally less than 1.0 μs for a repetition rate *f*_r_ lower than 50 kHz. As a result, the gate-open time *T*_go_ was usually controlled at 1.0 μs for a repetition rate *f*_r_ lower than 50 kHz. However, the inversion population decreases with an increase in the repetition rate *f*_r_, causing the buildup time *T*_bu_ to grow. In addition, the effective pumping time (*T*_p_ − *T*_go_) during the low-Q cycle starts to be closer to the buildup time *T*_bu_ for *f*_r_ higher than 100 kHz. The optimal value for the gate-open time *T*_go_ for a repetition rate *f*_r_ exceeding 100 kHz is determined by the conditions of *T*_go_ > *T*_bu_ as well as maximizing (*T*_p_ − *T*_go_). In brief, the gate-open time *T*_go_ leads to a significant influence on the performance of the acousto-optic Q-switched laser for a repetition rate *f*_r_ higher than 100 kHz. Even so, the experimental results revealed that when the gate-on time was substantially shorter than 0.5 μs, the peak-to-peak stability started to become worse because we came close to violating the criterion of *T*_go_ > *T*_bu_. Consequently, we explored the influence of the gate-open time on performance in this work by using *T*_go_ = 0.5 μs, 0.7 μs, and 1.0 μs to make a detailed comparison.

**Figure 4:**
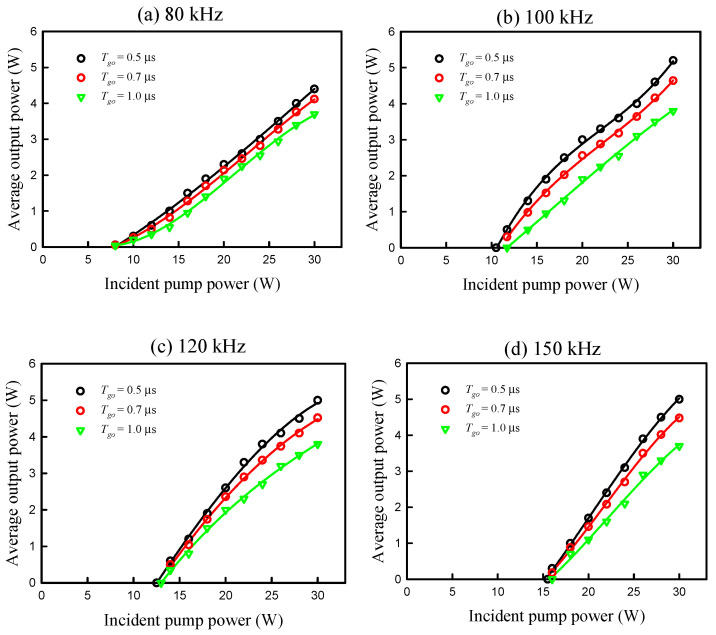
Data measured at four different repetition rates of *f_r_* = 80, 100, 120, and 150 kHz for average output power versus incident pump power for both cases of *T_go_* = 1.0 and 0.5 μs.

Figure 4 shows the experimental data measured at four different repetition rates of *f_r_* = 80, 100, 120, and 150 kHz for the average output power versus the incident pump power for the cases of *T_go_* = 1.0, 0.7, and 0.5 μs. The output efficiencies with *T_go_* = 0.5 μs can be seen to be obviously higher than those obtained with *T_go_* = 0.7 and 1.0 μs, especially for a repetition rate greater than 100 kHz. The lasing thresholds can be seen to increase with an increase in the repetition rate for all gate-open times. The experimental results revealed that the differences for the lasing thresholds between the gate-open times of 0.5 μs and 1.0 μs were not significant. To be brief, the overall efficiencies obtained with *T*_go_ = 0.5 μs were superior to the results obtained with *T*_go_ = 0.7 and 1.0 μs without conspicuously sacrificing the lasing thresholds. The enhancement of the output efficiency by shortening the gate-open time can be simply discussed as follows. For an incident pump power *P_in_* at a pump cycle *T_p_* = 1/*f_r_*, the initial inversion population *N_i_* can be expressed as

(3) $$N_{i}=\frac{P_{in}\tau_{f}}{h\nu_{p}}\left[1-e^{-(T_{p}-\;T_{go})/\tau_{f}}\right]+N_{f}$$

where *N_f_* is the final inversion population at the end of the previous pulse emission, τ*_f_* is the upper-level life time, and *hv_p_* is the pump photon energy. Under the condition of $(T_{p}-\;T_{go})<<\tau_{f}$, Equation (1) can be simplified as

(4) $$N_{i}=\frac{P_{in}(T_{p}-\;T_{go})}{h\nu_{p}}+N_{f}$$

It is clear that shortening the gate-open time *T*_go_ can effectively increase the initial inversion population *N_i_*. Since not only *N_i_* but also *N_f_* are raised by shortening the gate-open time *T*_go_, the enhancement of the output efficiency can be considerably grown from 10% to 30% when the repetition rate increases from 80 kHz to 150 kHz. It is worthwhile to discuss the dependence of optimal *T*_go_ on the pulse repetition rate. The experimental results revealed that the shortest values for *T*_go_ at the maximum pump power of 30 W were 3.5, 3.8, 4.2, and 4.5 μs for pulse repetition rates of 80, 100, 120, 150 kHz, respectively. The output power for the operation with the shortest *T*_go_ could reach a relative maximum. Nevertheless, the overall increase in the output power was not significant when the gate-open time *T*_go_ changed from 0.5 μs to the shortest values. Furthermore, the pulse train for the operation with the shortest *T*_go_ usually displays considerable fluctuation. In a compromise between output power and pulse stability, the optimal gate-open time *T*_go_ for a repetition rate between 80 and 150 kHz can be identified to be approximately 0.5 μs.

**Figure 5:**
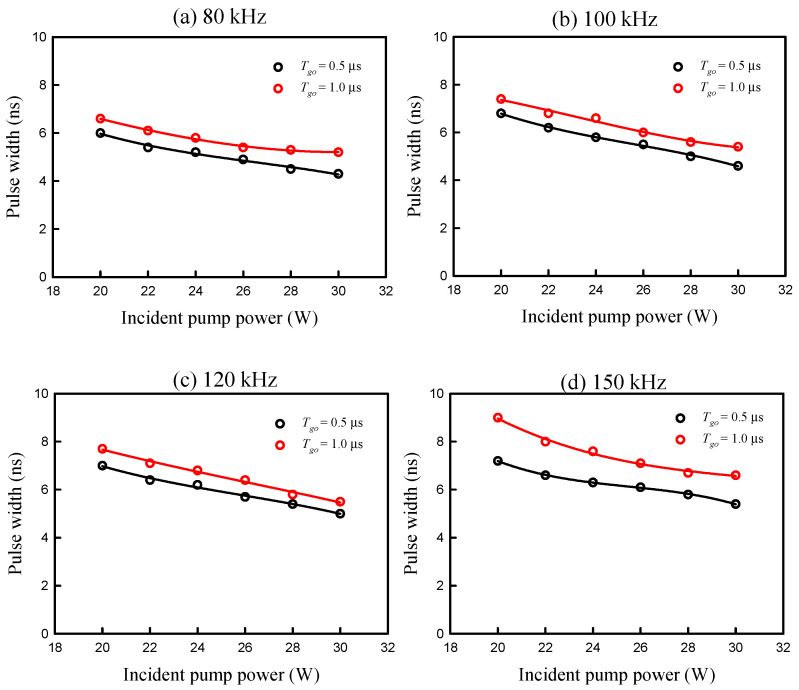
Experimental results for the pulse width versus the incident pump power obtained at four different repetition rates of *f_r_* = 80, 100, 120, and 150 kHz for both cases of *T_go_* = 1.0 and 0.5 μs.

**Figure 6:**
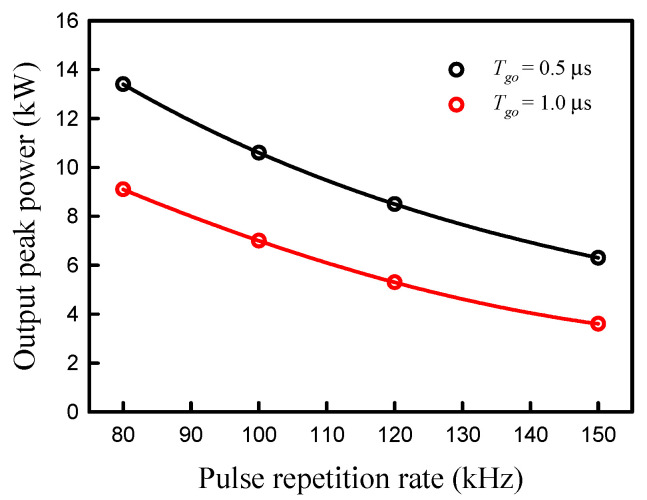
Calculated result for the output peak power versus the pulse repetition rare at the maximum pump power of 30 W for both cases of *T_go_* = 1.0 and 0.5 μs.

Figure 5 shows the experimental results for the pulse width versus the incident pump power obtained at four different repetition rates of *f_r_* = 80, 100, 120, and 150 kHz for both cases of *T_go_* = 1.0 and 0.5 μs. The pulse widths obtained with *T_go_* = 0.5 μs can be clearly seen to be shorter than those obtained with *T_go_* = 1.0 μs for four repetition rates. Furthermore, it was found that the pulse width increases with an increase in the repetition rate for both cases of *T_go_* = 1.0 and 0.5 μs. All these results agree quite well with the expectation that the higher the population inversion density, the shorter the pulse width. From the experimental results shown in Figure 4 and Figure 5, the output peak power was calculated as a function of the pulse repetition rate. Figure 6 shows the calculated result for the output peak power versus the pulse repetition rare at the maximum pump power of 30 W for both cases of *T_go_* = 1.0 and 0.5 μs. The output peak powers obtained with *T_go_* = 0.5 μs can be seen to be significantly greater than those obtained with *T_go_* = 1.0 μs. In addition, the output peak power can exceed 10 kW for a pulse repetition rate between 80 and 120 kHz for the case of *T_go_* = 0.5 μs. Even though the present output peak powers are not higher than those attained from the Q-switched Nd:YVO_4_/YVO_4_ eye-safe Raman laser [33], these data are the best results from the Nd:YVO_4_/KGW system. In addition, the conversion efficiency achieved in the present case is slightly higher than that obtained by the Nd:YVO_4_/YVO_4_ system [33].

**Figure 7:**
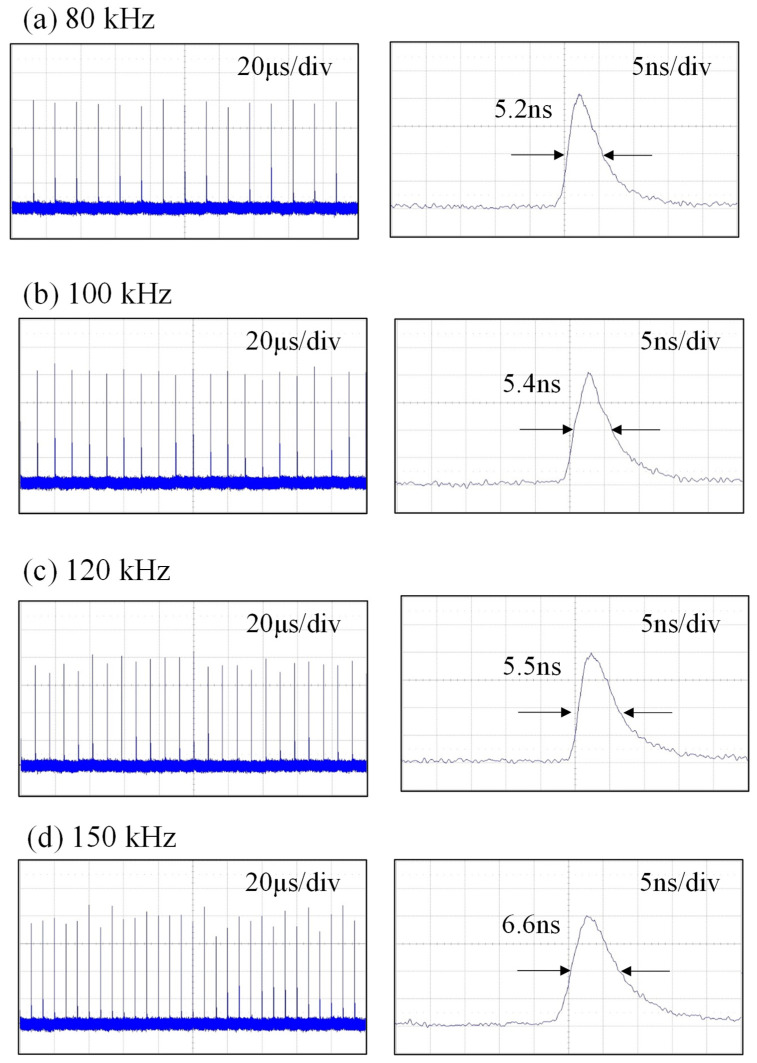
Temporal characteristics for pulse trains and single pulse shapes recorded at the maximum output powers for different repetition rates of 80, 100, 120, and 150 kHz with *T_go_* = 1.0 μs.

**Figure 8:**
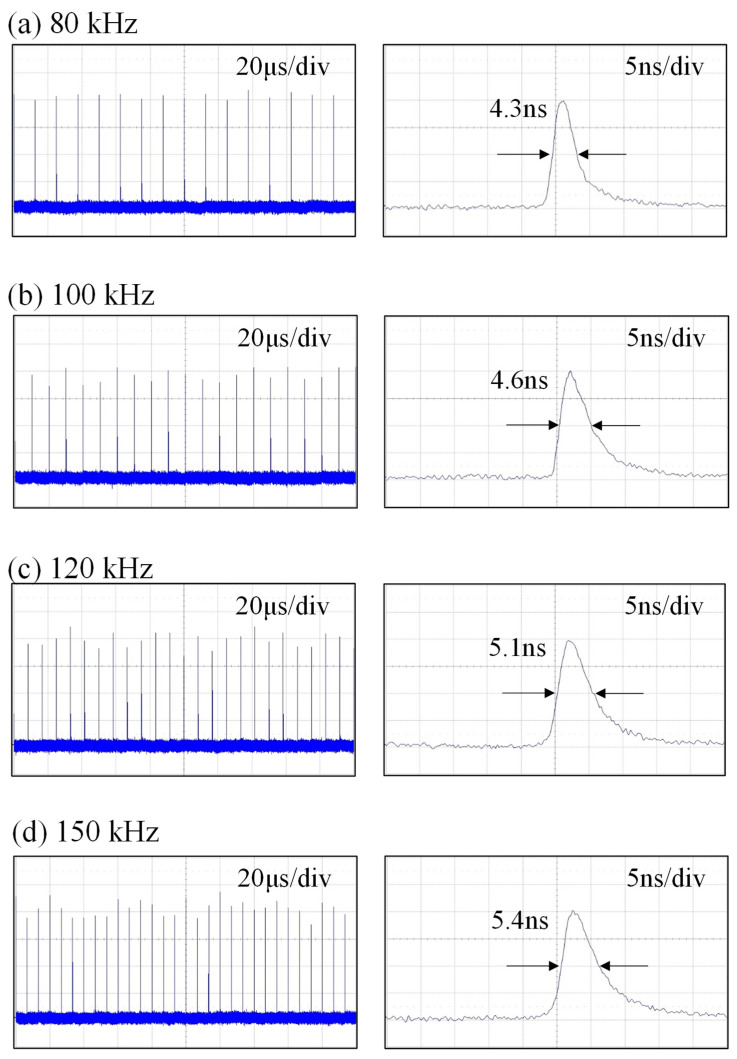
Temporal characteristics for pulse trains and single pulse shapes recorded at the maximum output powers for different repetition rates of 80, 100, 120, and 150 kHz with *T_go_* = 0.5 μs.

Figure 7 shows the temporal characteristics for pulse trains and single pulse shapes recorded at the maximum output powers for different repetition rates of 80, 100, 120, and 150 kHz with *T_go_* = 1.0 μs. The peak-to-peak amplitude stabilities were measured to be approximately ±5%, ±8%, ±12%, and ±15% for repetition rates of 80, 100, 120, and 150 kHz, respectively. In other words, the peak-to-peak fluctuation increases with an increase in the pulse repetition rate. Furthermore, there are almost no satellites to accompany the main output pulses, as seen in Figure 7. From the measured results, the average power fluctuations at the maximum output powers were found to be approximately ±5.0% for one hour. Figure 8 shows plots similar to Figure 7 for the case of *T_go_* = 0.5 μs. The overall performances for the temporal behaviors obtained with *T_go_* = 0.5 μs were found to be nearly the same as those obtained with *T_go_* = 1.0 μs.

The influence of output reflectivity on performance is also worthy of being discussed. The experimental results revealed that there were no significant differences between the output powers obtained with an output reflectivity of 90% and 80%. However, the output pulses attained with an output reflectivity of 90% were found to be easily accompanied by a few small satellites. On the other hand, the maximum output power obtained with an output reflectivity of 70% was found to be lower than that obtained with 80%. In addition, the maximum pulse repetition rate operated with an output reflectivity of 70% was found to be limited to 120 kHz. Therefore, the optimal output reflectivity can be identified to be near 80%.

**Figure 9:**
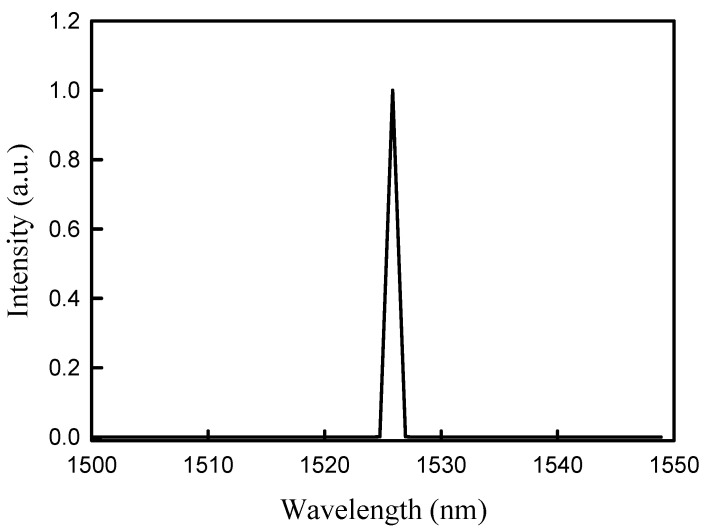
Wavelength spectrum of the output beam obtained at the maximum output power at a repetition rate of 150 kHz.

Figure 9 shows the wavelength spectrum of the output beam obtained at the maximum output power at a repetition rate of 150 kHz. The central wavelength is consistent with the expected value of 1526 nm. Finally, the knife-edge method was employed to evaluate the beam quality M^2^ factors for the Stokes output. The measured results revealed that the beam quality M^2^ factor considerably depends on the incident pump power but weakly on the pulse repetition rate. The measured beam quality M^2^ factor at the maximum output power was found to be approximately 1.8 ± 0.3 for a pulse repetition rate within 80–150 kHz. Furthermore, the influence of the gate-open time on the beam quality was found to be insignificant for *T_go_* > 0.45 μs. It is worthwhile to note that the residual output power for the fundamental wave was too low to measure the beam quality for the fundamental wave at 1342 nm.

## 4. Conclusions

In summary, we exploited the acousto-optic Q-switch to demonstrate a high-repetition-rate Nd:YVO_4_/KGW eye-safe Raman laser at 1526 nm. We explored the influence of the gate-open time on performance by using *T*_go_ = 0.5 μs and 1.0 μs to make a detailed comparison for repetition rates from 80 to 150 kHz. We employed a configuration of separate cavities for the fundamental and Stokes waves to shorten the pulse width. Under the optimal condition, the average output power can be greater than 5.0 W at a pump power of 30 W for a repetition rate within 100–150 kHz with *T*_go_ = 0.5 μs. Furthermore, the output peak power can be higher than 10 kW for a pulse repetition rate between 80 and 120 kHz. The overall optical-to-optical conversion efficiency is greater than 16.7%, slightly higher than that obtained by the Nd:YVO_4_/YVO_4_ system [33]. To the best of our knowledge, the conversion efficiency obtained here is the highest one for developing Nd:YVO_4_/KGW eye-safe Q-switched lasers.

## Data Availability

All data reported in the paper are presented in the main text. Any other data will be provided on request.
